# Reproductive health decision making among Ghanaian women

**DOI:** 10.1186/1742-4755-11-23

**Published:** 2014-03-15

**Authors:** Eugene Kofuor Maafo Darteh, David Teye Doku, Kobina Esia-Donkoh

**Affiliations:** 1 Department of Population and Health, University of Cape Coast, Private Mail Bag, University Post Office, Cape Coast, Ghana

## Abstract

**Introduction:**

Women’s reproductive health decision-making and choices, including engaging in sexual intercourse and condom use, are essential for good reproductive health. However, issues concerning sexual intercourse and condom use are shrouded in secrecy in many sub-Saharan African countries. This study investigates factors that affect decision making on engaging in sexual intercourse and use of condom among women aged 15–49.

**Method:**

A nationally representative sample (N = 3124) data collected in the 2008 Ghana Demographic and Health Survey was used. Multivariate logistic regression was used to study the association between women’s economic and socio-demographic characteristics and their decision making on engaging in sexual intercourse and use of condom.

**Results:**

One out of five women reported that they could not refuse their partners’ request for sexual intercourse while one out of four indicated that they could not demand the use of condoms by their partners. Women aged 35–49 were more likely to make decision on engaging in sexual intercourse (OR = 1.35) compared to those aged 15–24. Furthermore, the higher a woman’s education, the more likely that she would make decision regarding condom use. Also, if a woman had primary (OR = 1.37) or secondary (OR = 1.55) education, she is more likely to make decision regarding engaging in sexual intercourse compared to a woman who had no formal education. Compared to women in the Greater Accra region (the capital city region), women in the Western region (OR = 2.10), Central region (OR = 2.35), Brong Ahafo (OR = 1.70), Upper East (OR = 7.71) and Upper West (OR = 3.56) were more likely to make decision regarding the use of condom. Women who were in the richest, rich and middle wealth index categories were more likely to make decision regarding engaging in sexual intercourse as well as condom use compared to the poorest.

**Conclusion:**

Interventions and policies geared at empowering women to take charge of their reproductive health should focus particularly on women from less wealthy backgrounds and those with low educational attainments.

## Introduction

Over the years, the debate and research on women’s decision making in general and in sexual and reproductive health in particular have largely been genderised within power relations with men playing influential roles in women’s decision making. By extension, women are perceived to be less ‘empowered’ to take their own decision as a right. Guided by the expression of power framework [1], the paper draws insight from the ‘power to’ and ‘power within’ power expressions of the framework which explains one’s own self-worth and assertiveness based on one’s own personal characteristics, and one’s ability to act decisively.

Previous studies have investigated some aspects of women’s sexual and reproductive health decision making in sub-Saharan Africa [2-7]. In a study in selected African countries, Do & Kurimoto [4] found positive association between women empowerment score and contraceptive use in Ghana, Namibia, Uganda, Zambia and Zimbabwe. Also in Egypt, a study found an association between having some control in household matters and family planning decision making and contraceptive use [8]. The current study however, focuses on the factors that influence women’s reproductive health decision making.

The Ghana Demographic Health Surveys over the period have focused on trends of knowledge and use of family planning services. Notably among these are knowledge and use of family planning and fertility preferences. The 2008 Ghana Demographic and Health Survey shows that knowledge of at least one family planning method (contraceptive) is almost universal (98%) among Ghanaian women. However, the percentage of currently married women 15–49 currently using any method and any modern method of contraceptive declined from 25% to 24% and 19% to 17% respectively from 2003 to 2008 [9]. The report further reveals that there has been an increase in the percentage of women wanting no more children. The percentage was tripled in 1993 from 11% in 1979–80. The desire for more children has stabilised since 1993, with about the same proportion of women wanting no more children between 1998 and 2008 (36% - 37%). Same can be said about women’s ideal family size which declined noticeably from 5.9 to 4.7 from 1979–80 to 1993 but increased slightly in 2003 (4.8%) before declining again marginally in 2008 (4.6%). These findings signify a high unmet need for family planning among women in their reproductive period in the country. Using a nationally representative data this paper therefore, seeks to examine the factors that influence women’s reproductive health decision making regarding engaging in sexual intercourse and condom use.

### Theoretical and empirical issues

The concept of decision making has diverse connotations depending on the nature of the discourse being discussed, the intention of the user and the prevailing legal and global instruments. Literature available puts the concept into three perspectives. These are decision making as a process [10], as a choice [11], and as a right [12].

As a process, decision making goes through a number of stages, namely, identification of a problem, gathering data for analyses, developing alternative solutions, selecting an appropriate solution, implementing the solution and finally, evaluation [13]. Background characteristics such as information available and level of knowledge have potential influence on an individual’s ability to access information and act on them. Thus, the level of one’s education (as a measure of acquisition of information) is a potential influential factor on effective decision making. By extension, a woman’s decision making about her sexual and reproductive health will be effectively accomplished based on her exposure to knowledge. This argument is consistent with the Pred’s Behavioural Matrix [14]. Nevertheless, there are possibilities of other background characteristics such as religious affiliation and cultural inclinations that can have impact on the decision making process [15]. In sub-Saharan Africa in particular, social and cultural environments also affect women’s reproductive health decision making [2].

Women’s right to health including reproductive health was strongly affirmed by the International Conference on Population and Development (ICPD) in 1994 and the Fourth World Conference on Women in 1995 [16,17]. However, in most countries particularly those in sub-Saharan Africa, this affirmation has not been without controversies given the different socio-cultural contexts [3,18]. Similarly, choice is one of the cardinal principles enshrined in reproductive health policies worldwide [16]. For instance, the ICPD programme of action advocates for individuals to have the freedom to make reproductive health choices. Nevertheless, women’s autonomy is often restricted by economic, social and cultural circumstances prevailing in the region [2].

### Data and methods

This paper is based on data from the female file of the 2008 Ghana Demographic and Health Survey (GDHS). A total of 3124 females aged 15–49 randomly selected from rural and urban settlements are used for the analysis [9]. The respondents were asked if they could refuse to have sexual intercourse with their partners and whether they could ask their partners to use condoms. These two questions: women’s decision making on engaging in sexual intercourse and condom use were used to assess women’s reproductive health decision making. The response categories to these questions were: 1. Yes 2. No 3. Don’t Know/Not sure. These responses were re-coded into dichotomous variables [No and Don’t Know = 0 and Yes = 1]. The literature is not consistent on whether ‘no’ and ‘don’t know’ responses mean the same. Whilst some argue that ‘no’ and ‘don’t know’ mean different things and should therefore be analysed separately [19] others are of the view that they are similar and could be analysed together [20,21]. For the purpose of this paper we have subscribed to the second school of thought as there were fewer cases for the ‘don’t know’ category. This is consistent with a recent literature on sexual behaviour [20]. Explanatory variables included in the estimations were age, sex, educational level, marital status, wealth status, residence (urban–rural) and region of residence. Age was grouped using 10-year intervals (except the last category which ranged from 35 to 49 because those 45–49 were not many): 15–24, 25–34, 35–49. Four categories were created for level of education: no education, primary, secondary school and higher education. Place of residence was categorised into rural or urban. For region of residence, all ten administrative regions of Ghana were included in the analysis. To reflect the religious denomination of Ghana, group membership was used to categorise religion into: Catholic, Protestant (Anglican, Methodist Presbyterian), Pentecostal/Charismatic, Other Christians (spiritual churches), Muslim, Traditional/Spiritualist, No religion and Others. Wealth index was constructed from ownership of a variety of household items and categorised in quintiles poorest, poorer, middle, rich and richest. Ethnicity was categorised into Akan, Ga/Adangme, Ewe, Mole-Dagbani and Grussi, Gruma and Others. These independent variables are considered important in women’s reproductive health decision making in Ghana. These explanatory variables were used based on previous studies [6-8]. Logistic regression models were used to estimate the association between explanatory and dependent variables. Two logistic regression models were used to assess the relationship between the predictors of women’s decision making regarding sexual intercourse and condom use. In model I, we tested for the relationship between specific explanatory variables and each of the outcome variables separately. For model II, selected explanatory variables were used to examine the relationships between them and the outcomes variables. We conducted correlation analyses for the variables for which we suspected some correlations but the results showed weak and non-significant correlations (results not shown). STATA 11 (College Station, TX) statistical analytical tool was used for the analyses.

## Results

### Socio-demographic characteristics of respondents

The mean age for the women was 32.8 years. More than 21% of the women were aged between 25–19 years and about 3% were aged 13–19 years [Table 1]. Two-thirds of the women lived in rural areas. About 40% of the respondents had had secondary level education with about 34% indicating that they had no formal education. Forty-one per cent of the women were Akans and more than 2 in 10 of them were Mole-Dagbani. Majority of the respondents belonged to one religious grouping or the other. About a third were Pentecostal/Charismatic with another 15% being Catholics. A fourth of the women were considered to be among the poorest category with only 17% being among the richest. About 95% of the respondents were married (76%) or living together (19%). One out of five women could not refuse their partners request for sexual intercourse whiles 2% were not sure if they could. Again one out four indicated that they could not demand the use of condoms by their partner and 5% were not sure if they could.

**Table 1:** Socio-demographic characteristics of respondents

**Characteristics**	**Percentage**
Mean age	32.8
**Age of respondents**	**N = 3,124**
15-19	3.4
20-24	14.8
25-29	20.7
30-34	18.2
35-39	18.1
40-44	13.3
45-49	11.5
**Region**	**N = 3,124**
Western	9.12
Central	6.59
Greater Accra	11.62
Volta	9.92
Eastern	8.74
Ashanti	15.56
Brong Ahafo	8.87
Northern	11.78
Upper East	8.13
Upper West	9.67
**Place of residence**	**N = 3,124**
Rural	61.43
Urban	38.57
**Level of education**	**N = 3124**
No education	34.4
Primary	21.5
Secondary	40.5
Higher	3.3
**Ethnicity**	**N = 3124**
Akan	41.2
Ga/Dangme	5.6
Ewe	13.8
Mole-Dagbani	23.6
Other	15.9
Religion	**N = 3,124**
Catholic	14.2
Protestant	13.3
Pentecostal/charismatic	32.5
Other Christian	9.8
Moslem	17.7
Traditional/spiritualist	6.9
No religion	4.5
Other	0.3
**Wealth index**	**N = 3,124**
Poorest	25.6
Poorer	20.5
Middle	17.3
Richer	19.5
Richest	17.0
**Marital status**	**3124**
Married	75.6
Living together	18.8
Not living together	5.6
**Dependent variables**	
*Ability to refuse sex*	**N = 2950**
Yes	78.9
No	18.6
Not sure	2.5
*Ability to request condom use*	**N = 2950**
Yes	70.2
No	25.3
Not sure	4.5

Computed from the 2008 Ghana Demographic and Health Survey.

In Table 2, results from a logistic regression model on women’s decision making on sexual intercourse is presented. In model I, women aged 35 years and above were 1.3 times more likely to decide whether to have sex or not (p < .05) compared to those aged 15–24 years. The effect of a women’s age on her ability to take decisions on whether to have sex was 1.4 times (p < .05) compared to those aged 15–24 years in model II. Women in the Western region were less likely to decide whether to have sexual intercourse or not in model I (p < .001) compared to those in the Greater Accra region. On the other hand, women in the Central region were 1.8 times more likely to decide whether to have sexual intercourse or not (p < .05) compared to those from the Greater Accra region (see Table 2). The results in model I show that there is a significant relationship between place of residence and women’s ability to decide to have sexual intercourse with those residing in urban areas being 1.5 times more likely to decide whether to have sexual intercourse or not compared to those in rural areas (p < .001). The relationship between level of education and decision making on sexual intercourse was mainly positive and significant for models I and II. For instance, in model I, compared to those without formal education, women with primary education were about 1.4 times more likely to take decisions concerning sexual intercourse (p < .05). A similar pattern was found in model II where women with primary level education were 1.4 times more likely to take decisions concerning sexual intercourse (p < .05). Women with secondary level education were 1.7 times more likely to take decisions regarding whether to have sexual intercourse compared to those with no formal education (p < .001). However, the effect of education on women’s ability to take decisions regarding sexual intercourse reduced to 1.5 times in model II compared to those without formal education (p < .001) (see Table 2). The relationship between ethnicity and decision making on sexual intercourse were mainly negative and not statistically significant in both models except that women who belonged to the Mole-Dagbani ethnic group were about 0.70 (p < .05) and 0.46 (p < .001) times less likely to make decisions on sexual intercourse in models I and II respectively compared to the Akan ethnic group. Wealth status was positively and significantly related to women’s decision making on sexual intercourse in both models. For instance, whiles women in the middle on the wealth index were more than 2 times more likely to make decisions on sexual intercourse in model I compared to the poorest (p < .001), in model II, those in this category were about 1.8 times more likely to make decisions on sexual intercourse (p < .001) compared to the poorest on the wealth index.

**Table 2:** Logistic regression model showing the relationship between socio-demographic factors and women’s decision making on engaging in sexual intercourse

**Predictors**	**Model 1 Odds ratio**	**95% Confidence interval**	**Model 2 Odds ratio**	**95% Confidence interval**
**Age groups**				
15-24	Ref		Ref	
25-34	1.150	[0.89 - 1.48]	1.175	[0.91 – 1.54]
35-49	1.318**	[1.02 – 1.70]	1.353**	[1.05 – 1.75]
**Region**				
Greater Accra	Ref		Ref	
Western	0.506***	[0.33 – 0.76]	0.725	[0.46 – 1.14]
Central	1.582	[0.90 – 2.78]	1.824**	[1.03 – 3.24]
Volta	1.456	[0.90 – 2.37]	1.485	[0.88 – 2.50]
Eastern	0.758	[0.49 – 1.18]	0.923	[0.58 – 1.46]
Ashanti	0.450***	[0.31 – 0.65]	0.596**	[0.40 – 0.90]
Brong Ahafo	0.681	[0.44 -1.05]	1.182	[0.73 – 1.92]
Northern	0.640**	[0.43 – 0.95]	2.088***	[1.25– 3.50]
Upper East	0.815	[0.52 – 1.28]	2.986***	[1.67 – 5.35]
Upper West	0.728	[0.48 – 1.11]	2.504***	[1.44 – 4.36]
**Place of residence**				
Rural	Ref		Ref	
Urban	1.462***	[1.20 – 1.78]	1.175	[0.89 – 1.56]
**Level of education**				
No education	Ref		Ref	
Primary	1.370**	[1.07 – 1.75]	1.374**	[1.05 – 1.80]
Secondary	1.740***	[1.40 – 2.16]	1.546***	[1.16 – 2.05]
Higher	1.786**	[1.01 – 3.14]	1.096	[0.61 – 1.97]
**Religion**				
Catholic	Ref		Ref.	
Anglican	1.951	[0.44 – 8.53]	2.143	[0.48 – 9.52]
Methodist	0.763	[0.48 – 1.20]	0.742	[0.46 – 1.19]
Presbyterian	1.097	[0.69 – 1.75]	1.034	[0.64 – 1.67]
Pentecostal/charismatic	0.940	[0.69 – 1.27]	0.865	[0.63 – 1.19]
Other Christian	0.812	[0.55 – 1.20]	0.771	[0.51 – 1.16]
Moslem	0.812	[0.58 – 1.13]	0.861	[0.56 – 1.23]
Traditional/spiritualist	0.704	[0.46 – 1.06]	0.706	[0.46 – 1.09]
No religion	0.438***	[0.28 – 0.68]	0.518***	[0.33 – 0.82]
Other	1.365	[0.16 – 11.27]	1.442	[0.17 – 12.20]
**Ethnicity**				
Akan	Ref		Ref.	
Ga/Dangme	0.866	[0.57 – 1.32]	0.803	[0.51 – 1.27]
Ewe	1.343	[0.97 – 1.86]	0.959	[0.64 – 1.43]
Mole-Dagbani	0.703**	[0.56 – 0.88]	0.455***	[0.31 – 0.67]
Grussi	1.311	[0.80 – 2.15]	0.861	[0.47 – 1.57]
Gruma	0.718	[0.47 – 1.09]	0.812	[0.48 – 1.36]
Other	0.828	[0.56 – 1.22]	0.737	[0.47 – 1.15]
**Wealth status**				
Poorest	Ref		Ref	
Poorer	1.249	[0.97 – 1.61]	1.283	[0.97 – 1.70]
Middle	2.099***	[1.54 – 2.85]	1.774***	[1.25 – 2.52]
Richer	1.750***	[1.32 – 2.31]	1.589**	[1.09 – 2.32]
Richest	1.854***	[1.38 – 2.48]	1.603**	[1.02 – 2.52]

Computed from the 2008 Ghana Demographic and Health Survey.

***p < .001 **p < .05.

Table 3 presents, results from a logistic regression model on women’s decision making on condom use. Model I shows that, compared to women in the Greater Accra region, women in the Ashanti region (OR = 0.48) and Northern region (OR = 32) were less likely to make decisions on condom use. The findings on region and women’s decision making on condom use was however different in model II. Compared to women in the Greater Accra region, women in the Western region (OR = 2.10), Central region (OR = 2.35), Brong Ahafo (OR = 1.70), Upper East (OR = 7.71) and Upper West (OR = 3.56) were more likely to make decision on condom use. There was a positive and significant relationship between place of residence and decision making on condom use such that women in urban areas were 1.5 times more likely to make decisions on condom use compared to those in rural areas (p < .001). Furthermore, the results for education shows that in both models, the higher a woman’s educational level, the higher the likelihood that she would make decision concerning condom use compared to those without any formal education. Woman’s ethnicity was related to her condom use decision making in both models I and II among Ga/Dangmes. In model I, for example, women belonging to this ethnic group were 1.9 times more likely to make decisions regarding condom use compared to Akans (p < .05). On the other hand, this effect increased in model II among the Ga/Dangmes (Table 3). They were 2.0 times more likely to make decision concerning condom use compared to the Akans (p < .001). There was a positive and significant relationship between wealth status and women’s ability to make decisions concerning condom use in both models. Women who were in middle (OR = 1.18), richer (OR = 1.80), richer (OR = 2.15) and richest (OR = 2.31) were more likely to make decisions on condom use compared to the poorest (Model I). In model II on the other hand, those who were middle (OR = 1.77), richer (OR = 1.59) and richest (OR = 1.60) on the wealth index had higher probability of making decision on condom use compared to the poorest (Table 3).

**Table 3:** Logistic regression model on predictors of women’s decision making on condom use

**Predictors**	**Model 1 Odds ratio**	**95% Confidence interval**	**Model 2 Odds ratio**	**95% Confidence interval**
**Age groups**				
15-24	Ref		Ref	
25-34	1.119	0.88 - 1.42	1.064	[0.83 – 1.36]
35-49	0.981	0.78 - 1.23	0.984	[0.77 – 1.25]
**Region**				
Greater Accra	Ref		Ref	
Western	1.020	0.68 - 1.52	2.101***	[1.33 – 3.32]
Central	1.303	0.82 - 2.06	2.350***	[1.41 – 3.91]
Volta	0.885	0.60 - 1.30	0.764	[0.48 – 1.21]
Eastern	1.275	0.84 - 1.94	1.343	[0.86 – 2.09]
Ashanti	0.484***	0.34 - 0.67	0.728	[0.50 – 1.07]
Brong Ahafo	0.805	0.55 - 1.19	1.703**	[1.08 – 2.69]
Northern	0.320***	0.23 - 0.45	1.163	[0.73 – 1.86]
Upper East	1.516	0.97 - 2.36	7.710***	[4.31 – 13.83]
Upper West	0.801	0.55 - 1.16	3.564***	[2.11 – 6.02]
**Place of residence**				
Rural	Ref		Ref	
Urban	1.527***	1.28 - 1.82	1.063	[0.82 – 1.37]
**Level of education**				
No education	Ref		Ref	
Primary	1.556***	1.25 - 1.93	1.573***	[1.23 – 2.014]
Secondary	2.757***	2.26 - 3.36	2.576***	[1.99 – 3.34]
Higher	4.651***	2.46 - 8.80	4.267***	[2.18 – 8.34]
**Religion**				
Catholic	Ref		Ref.	
Anglican	3.086	0.71 -13.42	2.963	[0.65 – 13.54]
Methodist	0.980	0.64 - 1.49	0.913	[0.59 – 1.42]
Presbyterian	1.173	0.78 - 1.77	1.040	[0.68 – 1.59]
Pentecostal/charismatic	1.201	0.92 - 1.57	1.154	[0.86 – 1.54]
Other christian	0.961	0.68 - 1.36	1.030	[0.71– 1.50]
Moslem	0.690	0.52 - 0.92	1.369	[0.98 – 1.91]
Traditional/spiritualist	0.552	0.39 - 0.79	0.876	[0.59 – 1.31]
No religion	0.438	0.29 - 0.66	0.738	[0.48 – 1.14]
Other	Omitted		Omitted	
**Ethnicity**				
Akan	Ref		Ref.	
Ga/Dangme	1.902**	1.18 - 3.07	2.022***	[1.24 – 3.30]
Ewe	1.140	0.86 - 1.51	1.456**	[0.100 – 2.12]
Mole-Dagbani	0.659***	0.53 - 0.81	0.560**	[0.41 – 0.87]
Grussi	1.618**	1.01 - 2.60	1.095	[0.62 – 1.93]
Gruma	0.297***	0.21 - 0.42	0.675	[0.42 – 1.08]
Other	0.561***	0.40 - 0.78	0.639**	[0.43 – 0.96]
**Wealth status**				
Poorest	Ref		Ref	
Poorer	1.177***	0.94 - 1.48	1.206	[0.93 – 1.57]
Middle	1.801***	1.39 - 2.34	1.507**	[1.10 – 2.06]
Richer	2.146***	1.66 - 2.78	1.859***	[1.30 – 2.65]
Richest	2.308***	1.76 - 3.02	1.812***	[1.19 – 2.75]

Computed from the 2008 Ghana Demographic and Health Survey.

***p < .001 **p < .05.

## Discussion

The paper sought to examine the factors that influence women’s decision making in sexual and reproduction health issues. Decisions regarding whether to have sex and use condoms were used as measures for sexual and reproductive health decision making. This study found that one-fifth of Ghanaian women could not refuse their partners’ request for sexual intercourse whiles one out of four could not demand the use of condoms by their partners. Age and education were associated with decision making on engaging in sexual intercourse. Furthermore, the higher a woman’s education, the more likely that she would make decision regarding condom use. Compared to women in the Greater Accra region (the capital city region), women in the Western region, Central region, Brong Ahafo, Upper East and Upper West were more likely to make decision on condom use. Furthermore, women who were in the richest, rich and middle wealth index categories were more likely to make decisions on engaging in sexual intercourse as well as condom use compared to the poorest.

This study found that older women were more likely to make decision on engaging in sexual intercourse compared to the younger ones. Studies have shown that being a young woman in marriage or early marriage may result in powerlessness and difficulty in reproductive health decision making including condom use [22]. In Ghana and in most African countries, age is very important in that younger persons are expected to respect the older ones and arguing or engaging in intense discussions with an older person is frowned on. Besides, there is evidence of age mixing among couples in such a way that the woman is almost always younger than the man [9]. This cultural phenomenon may prevent women from negotiating for sexual intercourse or the use of condoms and other contraceptives.

Regional differences in the decision making on both engaging in sexual intercourse and condom use were found. Interestingly, the regions where women had higher likelihood of making decision regarding engaging in sexual intercourse compared to those living in the Greater Accra region were also the regions where women were more likely to making decision regarding condom use. The only exception was that women in the Northern region had higher chances of making decision on engaging in sexual intercourse compared to those in the Greater Accra region. Regional differences exist in Ghana by level of education, development and poverty [9]. The Northern, Upper East and Upper West regions are the least in terms of development and the poorest as well. Apart from these differences, there are also enormous cultural differences regarding sexual and reproductive health regionally. All these could account for the disparities in decision making regarding engaging in sexual intercourse and condom use found in this study. Future studies should explore the impact of, particularly, the cultural specificity in relation to reproductive health decision making in general. Such studies would clarify our understanding on the phenomenon.

Some studies seem to suggest that education is positively associated with reproductive health decision making [13,23]. Consistent with these previous studies, this study found that the higher a woman’s education, the more likely that she would make decision regarding condom use. Similarly, we found that if a woman had primary or secondary education, she is more likely to make decision regarding in sexual intercourse compared to a woman who had no formal education. Education does not only provide information vital for decision making but also self-confidence, self-reliance, empowerment and autonomy. All of these benefits of education are vital in decision making regarding one’s health and health behaviour. Indeed, education nurtures and consolidates the stages of decision making process [13].

Over all, no clear relationship between religion and ethnicity on decision making on engaging in sexual intercourse and condom use was found, except that women with no religion (OR = 0.52) were less likely to make decision on engaging in sexual intercourse compared to those of the Catholic faith while women of the Islamic faith were more likely to make decision regarding condom use, although at borderline significance.

Richest, richer and middle women as per wealth index were more likely to make decision on engaging in sexual intercourse as well as condom use compared to those in the poorest category. Wealth in its self may represent power. It may lead to independence, autonomy and may be related to education. For women in a developing country like Ghana where majority live in poverty, wealth may be very important in boosting the confidence and self-esteem of women, and may consequently give them the autonomy to make decision regarding reproductive health choices. The relationship between wealth index and decision making on engaging in sexual intercourse and condom use suggests that reduction in poverty or improving the living conditions of women would go a long way to improve their reproductive health decision making and hence their health.

This study is limited by its cross-sectional nature and hence causal inferences cannot be made. The variables used were self-reported and thus subject to the desirability or otherwise of the respondents. The nature of the data made it impossible to discuss gender relations and their effect on women’s reproductive health decision making. Despite the above limitations, the study has compelling strengths. First, the large sample size gave the study sufficient power. Moreover, the representativeness of the sampling strategy as well as the nationwide nature of the data boosts the study’s generalizability to other settings.

In conclusion, age, region of residence, level of education, ethnicity and wealth were found to be associated with Ghanaian women’s decision regarding engaging in sexual intercourse and condom use. This finding contributes to the discourse on women’s reproductive health decision making and adds to the scarce literature on sexual and reproductive health issues in African countries and Ghana in particular. Interventions and policies geared towards empowering women to take charge of their reproductive health should focus particularly on women from less wealthy backgrounds and those with low educational attainments. Future studies should explore the cultural specificity of decision making on engaging in sexual intercourse and condom as well as women’s decision making on reproductive health choices in general.

## Competing interests

The authors declare that they have no competing interest.

## Authors’ contributions

EKMD conceived the study. EKMD and DTD drafted the first version of the manuscript. EKMD, DTD and KED revised the manuscript for important intellectual content and gave consent for the version to be published. All authors have read and approved the final manuscript.
